# Daily Step Counts Before and After the COVID-19 Pandemic Among All of Us Research Participants

**DOI:** 10.1001/jamanetworkopen.2023.3526

**Published:** 2023-03-20

**Authors:** Stacy Desine, Hiral Master, Jeffrey Annis, Andrew Hughes, Dan M. Roden, Paul A. Harris, Evan L. Brittain

**Affiliations:** 1 Division of Cardiovascular Medicine, Vanderbilt University Medical Center, Nashville, Tennessee; 2 Vanderbilt Institute of Clinical and Translational Research, Vanderbilt University Medical Center, Nashville, Tennessee; 3 Department of Medicine and Biomedical Informatics, Vanderbilt University Medical Center, Nashville, Tennessee; 4 Departments of Biomedical Informatics, Biomedical Engineering and Biostatistics, Vanderbilt University Medical Center, Nashville, Tennessee

## Abstract

This cohort study of US adults examines changes in physical activity following the onset of the COVID-19 pandemic.

## Introduction

The COVID-19 outbreak had a global impact on physical, mental, and social health. Data collected early in the pandemic suggested a general decline in step counts worldwide,^1^ but factors contributing to reduced activity have not been identified.

This study aimed to examine whether COVID-19 was associated with daily activity among All of Us (AOU) research program^2^ participants. We hypothesized that a decline in activity across the US would persist after most social distancing recommendations were relaxed, and that socioeconomic factors and mental health status would continue to affect reduced activity.

## Methods

Using AOU Research Program Controlled Tier data set (released June 2022), this cohort study included participants who wore a digital device tracking physical activity (Fitbit Inc) for at least 10 days each month (eMethods in Supplement 1). Daily steps (averaged monthly) were examined over 4 years from January 2018 through December 2021. Counterfactual analysis based on 2 years of activity data preceding COVID-19 was used to estimate postpandemic steps. Differences in observed and estimated post–COVID-19 daily steps were examined using Wilcoxon rank sum test with continuity correction.

Surveys were used to assess sociodemographic factors, mental health, location, and deprivation index at the time of enrollment.^3^ Linear mixed models examined associations of COVID-19 vaccine, comorbidities, sociodemographic, and mental health factors with differences in observed and estimated post–COVID-19 daily steps (eMethods in Supplement 1).

In this study, only the authorized authors who completed AOU Responsible Conduct of Research training accessed the deidentified data from the Researcher Workbench (a secured cloud-based platform). Because the authors were not directly involved with the participants, institutional review board review and the requirement for informed consent was exempted. This study followed the Strengthening the Reporting of Observational Studies in Epidemiology (STROBE) reporting guideline. Analysis was conducted using R version 4.2.2 (R Project for Statistical Computing). The threshold for significance was *P* < .05 determined in 2-sided tests.

## Results

We analyzed data from 5443 (3903 [71.7%] female; 257 [4.7%] Black, 4681 [86.0%] White, 505 [9.3%] other; median [IQR] age, 53 [38-64] years) participants with valid Fitbit data for at least 6 months pre- and post–COVID-19 (Table). Median (IQR) observed daily step counts pre–COVID-19 (ranged from January 1, 2018, to January 31, 2020) and post–COVID-19 (ranged from June 1, 2020, to December 31, 2021) were 7808 (5923-10 108) steps and 7089 (5101-9740) steps, respectively. The counterfactual model estimated participants walked 575 (95% CI, 521-629) fewer steps per day post–COVID-19 compared with observed daily steps (*P* < .001; Figure, A). The difference between observed and estimated post–COVID-19 steps was significantly explained by younger age (β = −243 per 10-year decrease; *P* < .001), Northeast region compared with other regions (Northeast vs others, β = −288, *P* < .001), and higher deprivation index (β = −477 per 0.1 increment; *P* < .001) (Figure, B). Post–COVID-19 step counts were also explained by COVID-19 vaccination status (vaccinated vs unvaccinated: β = 48; *P* < .01), depression (β = −36 per 1 score increment; *P* < .01), and psychological stress (β = −13 per 1 score increment; *P* < .01) (Figure, C). We found no association between reduced step counts and sex or comorbidities such obesity, diabetes, coronary artery disease, hypertension or cancer.

**Table.** Demographic Characteristics

Characteristics	Participants, No. (%) (N = 5443)
Age, mean (IQR)	53.5 (38.50-63.50)
Sex at birth	
Female	3903 (71.7)
Male	1540 (28.3)
Race	
White	4681 (86.0)
Black	257 (4.7)
Other^a^	505 (9.3)
Geographic location	
Northeast	873 (16.0)
Midwest	2044 (37.6)
South	1174 (21.6)
West	1352 (24.8)
Median income, mean (IQR), $	61 193 (55 344-74 163)
Deprivation index, mean (IQR)	0.30 (0.28-0.34)
Impact of Event Scale score, mean (IQR)^b^	1.22 (0.75-1.83)
Patient Health Questionnaire score, mean (IQR)^b^	2.00 (1.00-4.00)
Perceived Stress Scale Score, mean (IQR)^b^	11.50 (6.50-18.00)
Documented COVID-19 infection during entire study period	100 (1.8)
≥1 Dose of COVID-19 vaccine during entire study period	2959 (54.4)
Obesity	666 (12.2)
Diabetes	180 (3.3)
Coronary artery disease	175 (3.2)
Hypertension	2053 (37.7)
Cancer	1240 (22.8)

^a^ Other included participants who self-reported as Asian, more than 1 race or ethnicity, did not answer or skipped, or preferred not to answer.

^b^ Total participants providing data included 3072 (56.4%) for the Impact of Events Scale, 2384 (43.8%) for the Patient Health Questionnaire, and 3056 (56.1%) for the Perceived Stress Scale.

**Figure.**
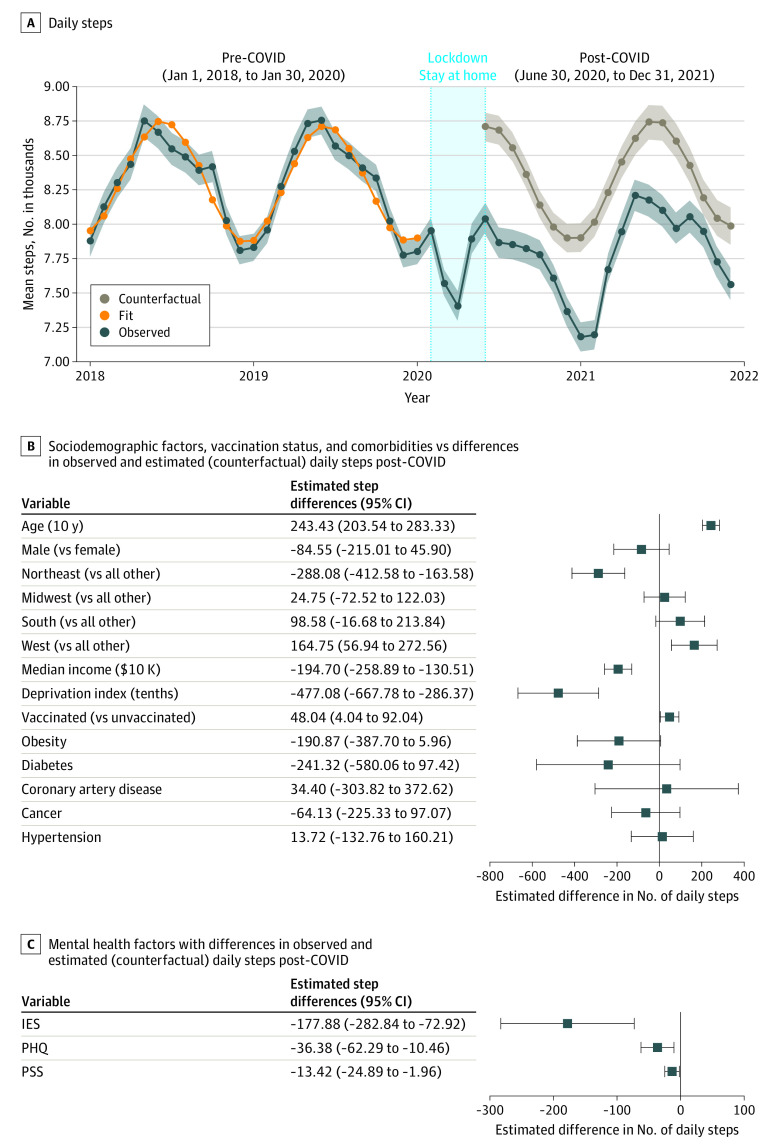
Impact of the COVID-19 Pandemic, Sociodemographics, and Mental Health Factors on Average Daily Steps From 2018-2021 In panel A, oscillation and dips potentially related to seasonal variation in daily steps counts could be observed in both pre–COVID-19 and post–COVID-19 phases. In panel B, analysis was conducted using a cosinor linear mixed-effects model. In panel C, models were adjusted for age, sex, deprivation index, median income, and region. IES indicates Impact of Events Scale; PHQ, Patient Health Questionnaire; PSS, Perceived Stress Scale.

## Discussion

These findings suggest a consistent, widespread, and significant decline in activity following the onset of COVID-19 in the US. Vulnerable populations, including individuals at a lower socioeconomic status and those reporting worse mental health in the early COVID-19 period, were at the highest risk of reduced activity. We found a statistically significant decline in daily step counts that persisted even after most COVID-19–related restrictions were relaxed, suggesting COVID-19 affected long-term behavioral choices. Currently, it is unknown whether this reduction is steps is clinically meaningful over time. Any meaningful difference is likely dependent on baseline activity, age, and other patient-level factors. Our prior work in the AOU cohort suggests that modestly lower step counts over a long period could have a substantial contribution to long-term disease risk.^4^

While our data include some selection bias (eg, majority were White, young, and active and only included participants who owned and reported valid tracking data), this finding extends the work of prior studies showing an early reduction in activity.^1,5^ We also imposed relatively stringent criteria for a valid tracking day, which could have biased our results toward more active individuals. The 2% of our cohort with prior COVID-19 infection likely underestimated the true prevalence as it did not include positive home tests. Despite these limitations, our study showed COVID-19 to be associated with decreased physical activity, which may have implications for future cardiometabolic risk with the potential to exacerbate health disparities related to socioeconomic status and mental health.

## Notes

Supplement 1.
eMethods.

Supplement 2.
Data Sharing Statement
