# Supplementary material for: Daily Step Counts Before and After the COVID-19 Pandemic Among All of Us Research Participants
Source: JAMA Netw Open. 2023 Mar 20;6(3):e233526. doi: 10.1001/jamanetworkopen.2023.3526 (PMC10028484; doi:10.1001/jamanetworkopen.2023.3526)
Supplement: Supplement 1. — eMethods. [file jamanetwopen-e233526-s001.pdf]

Supplemental Online Content

Desine S, Master H, Annis J, et al. Daily step counts before and after the COVID-19 pandemic among All of Us research participants. *JAMA Netw Open.* 2023;6(3):e233526. doi:10.1001/jamanetworkopen.2023.3526

eMethods.

This supplemental material has been provided by the authors to give readers additional information about their work.

© 2023 Desine S et al. *JAMA Network Open.*

eMethods

Participants:

In this study, participants who provided primary consent to be part of the *All of Us* Research Program and share EHR data had an opportunity to provide their Fitbit data under the Bring Your Own Device program. Therefore, participants who owned Fitbit device were able to contribute their Fitbit data since the time they created Fitbit account. In this study, only the authorized authors who completed *All of Us* Responsible Conduct of Research training accessed the deidentified data from the Researcher Workbench (a secured cloud-based platform). Since the authors were not directly involved with the participants, Institutional Review Board review was exempted.

Outcomes:

Fitbit data was reported as daily step counts over the monitoring period. Daily steps were averaged on a monthly basis for each participant over 4 years from January 2018 to December 2021. Daily steps were then examined from January 1, 2018 to January 31, 2020 (pre-COVID) and from June 1, 2020 to December 31, 2021 (post-COVID). Daily step counts during the lockdown period, defined as the timeframe from February 1, 2020 to May 31, 2020,) when maximal restriction strategies (e.g., stay at home) were imposed, were excluded from the analysis. .

Independent variables:

Age, sex, geography, and median income were assessed using the *All of Us* Basics Survey at the time of enrollment. Deprivation index was sourced from the U.S. Census American Community Survey via a three digit zip code linkage. A higher score on the deprivation index (range 0 to 1)

© 2023 Desine S et al. *JAMA Network Open.*

indicates higher deprivation in areas such as standard of living, health, education, economic security, housing quality, and neighborhood quality. Obesity, DM (diabetes mellitus), and CAD (coronary artery disease), Cancer, HTN (hypertension), vaccination status were defined using electronic health records. Responses on COVID-19 participant Experience surveys[4] were averaged over the months of May, June, and July 2020 to assess mental health factors such as impairments from post-traumatic stress disorder, depression, and psychological stress. EHRs were used to identify COVID-19 cases and vaccination status over the entire study period. Mental health factors were assessed using Impact of events scale (IES), Patient health questionnaire (PHQ) and Perceived Stress Scale (PSS). Higher scores on the IES score (ranges from 0 to 4), PHQ (ranges from 0 to 27), and PSS (ranges from 0 to 40) indicate higher levels of impairment from post-traumatic stress disorder, severe depression, and psychological stress, respectively.

Analysis:

Seasonal trends were accounted for with a cosinor model that describes a sinusoidal curve via a linear combination of a sin and cosine term:

$$x = \cos\left(\frac{2\pi m}{\tau}\right),$$

$$z = \sin\left(\frac{2\pi m}{\tau}\right),$$

where $m = \{1, 2, 3, \ldots 12\}$ is the ordered month of the year and τ is a fixed value representing the period (we let τ = 12). These two terms, in addition to a term to model linear changes in steps over time, were entered into a linear mixed-effects model (as both fixed and random effects) that

© 2023 Desine S et al. *JAMA Network Open.*

was fit to the pre-COVID step count data. Post-COVID predictions were then generated using the fitted model. The differences between the observed and predicted step counts were then modeled using a subsequent cosinor linear mixed-effects model with covariates introduced as fixed effects to measure their effect on the predicted change in step counts (with τ=6 for the best fit). Modeled differences between observed and predicted was calculated as observed minus predicted where a negative value indicates a decrease in the predicted step counts in the post-COVID era. These included deprivation index, median income, age, sex, vaccination status and region in addition to baseline statuses for hypertension, obesity, cancer, diabetes, and CAD. We then fit three additional linear mixed-effects models with IES, PHQ, PSS as fixed effects, each entered in a separate model (all adjusted for age, sex, deprivation index, median income, and region). Modeling was done separately for each survey-type to avoid a reduction in the sample size due to high amounts of missingness in survey responses (missingness in IES, PHQ, PSS were 44%, 56% and 44%, respectively).

© 2023 Desine S et al. *JAMA Network Open.*
