# Supplementary material for: Daily Step Counts Before and After the COVID-19 Pandemic Among All of Us Research Participants
Source: JAMA Netw Open. 2023 Mar 20;6(3):e233526. doi: 10.1001/jamanetworkopen.2023.3526 (PMC10028484; doi:10.1001/jamanetworkopen.2023.3526)
Supplement: Supplement 2. — Data Sharing Statement [file jamanetwopen-e233526-s002.pdf]

Data Sharing Statement

Desine. Daily Step Counts Before and After the COVID-19 Pandemic Among All of Us Research Participants. *JAMA Netw Open*. Published March 20, 2023. doi:10.1001/jamanetworkopen.2023.3526

Data
Data available: No

Additional Information
Explanation for why data not available: Not permitted by the All of Us Program
